# Eye Region Surface Temperature and Corticosterone Response to Acute Stress in a High-Arctic Seabird, the Little Auk

**DOI:** 10.3390/ani12040499

**Published:** 2022-02-17

**Authors:** Dariusz Jakubas, Katarzyna Wojczulanis-Jakubas, Antoine Grissot, Marion Devogel, Martyna Cendrowska, Olivier Chastel

**Affiliations:** 1 Department of Vertebrate Ecology and Zoology, Faculty of Biology, University of Gdańsk, 80-308 Gdańsk, Poland; biokwj@univ.gda.pl (K.W.-J.); antoine.grissot@hotmail.fr (A.G.); marion.devogel.v@gmail.com (M.D.); martynacendrowska16@gmail.com (M.C.); 2 Centre d’Etudes Biologiques de Chizé (CEBC), UMR 7372—CNRS & Université de la Rochelle, 79360 Villiers-en-Bois, France; olivier.chastel@cebc.cnrs.fr

**Keywords:** acute stress, body surface temperature, hormonal stress response, thermal stress response

## Abstract

**Simple Summary:**

Thermography, which is a method of measuring the heat emitted by various regions of the body, may be useful in detection of a heat increase in response to stress in vertebrates. Using this method, we studied how body surface temperature in eye-region (TEYE) changes in response to short-term stress (capturing and handling) in a wild-living, medium-sized polar seabird, the Little Auk. To this end, we measured TEYE in birds twice: first time—just after their capturing and initial handling and then second—after 30 min of keeping them in a bag. To control birds’ stress response, at the same time we made thermography, and we also collected blood samples from all the individuals, to establish the level of corticosterone (CORT, stress hormone). We found that both TEYE and CORT increased in response to the experimental procedure, although the strength of the TEYE and CORT increase were not related to each other. This indicates that thermography is a good tool for detection of initiation of birds’ reaction to a stress, which may be further useful in other studies, e.g., where there is a need to establish birds’ stress response non-invasively.

**Abstract:**

Measuring changes in surface body temperature (specifically in eye-region) in vertebrates using infrared thermography is increasingly applied for detection of the stress reaction. Here we investigated the relationship between the eye-region temperature (TEYE; measured with infrared thermography), the corticosterone level in blood (CORT; stress indicator in birds), and some covariates (ambient temperature, humidity, and sex/body size) in a High-Arctic seabird, the Little Auk *Alle alle*. The birds responded to the capture-restrain protocol (blood sampling at the moment of capturing, and after 30 min of restrain) by a significant TEYE and CORT increase. However, the strength of the TEYE and CORT response to acute stress were not correlated. It confirms the results of a recent study on other species and all together indicates that infrared thermography is a useful, non-invasive measure of hypothalamic-pituitary-adrenal (HPA) axis reactivity under acute activation, but it might not be a suitable proxy for natural variation of circulating glucocorticoid levels.

## 1. Introduction

In the natural environment, animals are frequently exposed to short-term, acute stressors, such as predators or aggressive conspecifics. Reaction to these stressful situations helps animals to survive a stressful episode [1,2,3]. The physiological chain of the reaction is as follows: the hypothalamic-pituitary-adrenal axis (HPA) is the first to be activated after exposition to a stressor, and this results in glucocorticoid secretion, which in turn prepares the whole body to the active mode: “flight-or-fight,” which is a very adaptive response to handle the stressful situation. This “fight-or-flight” response is automatic and is also associated with an activation of the sympathetic-adrenal-medullary system (SAM) causing rapid release of catecholamines from the adrenal medulla [4]. Thus, both glucocorticoids and catecholamines mediate physiological and behavioral changes made to deal with the stressor [5]. Importantly, the stress response involves a number of physiological effects including vasoconstriction (narrowing of the blood vessels resulting in skin cooling). This is expressed by an increase in core body temperature, called stress-induced hyperthermia (SIH) [6,7]. SIH reduces blood transport from the warmer core to the cooler periphery resulting in a decrease in body surface temperature [8]. This thermal reaction is widespread amongst endotherms [9,10,11,12,13]. Thus, changes in body temperature, if measurable, may be a good indicator of physiological responses to short-term stressors [14].

Infrared thermography is an increasingly popular method being applied in wild-living animals. Since it provides useful information regarding heat emission it is used as a non-invasive technique for measuring the thermal stress response of living organisms (e.g., [15,16,17,18,19]). For that purpose, uninsulated, i.e., unfettered by insulatory keratinous tissues (i.e., feathers or leg scale), highly vascularized parts of bodies are considered; that can be, for example, an eye region (the periophthalmic ring) [8,15,16,18,19,20,21].

Examining the stress response of birds is important to investigate many important topics in avian ecology, such as the response to a food shortage, increased parental efforts, and climate change, to mention only a few. Behavioral observations or the measurement of glucocorticoids or/and leucocytes levels in blood are widely used methods to assess the stress response in birds [14,22]. However, if the first approach is relatively non-invasive, it is also subject to strong observer bias, while the second one, if more accurate, is invasive for the bird (due to blood sampling). The use of the infrared thermography is then to provide accurate and non-invasive measurement of the stress response in birds. A non-invasive technique not only improves avian welfare during the study but also greatly simplifies a field procedure, which otherwise is challenging, i.e., blood sampling in a strict time regime, to establish a baseline and stress-induced change in stress hormones’ levels. Although the number of studies using infrared thermography for the avian stress response is growing, the taxonomical range of the studied species is so far quite narrow, including few passerines (i.e., Blue Tit *Cyanistes caeruleus* [15,16,19], Great Tit *Parus major* [18], and Black-capped Chickadee *Poecile atricapillus* [17]), pigeons (i.e., Domestic Pigeon *Columbia livia domestica* [21]), and galliformes (i.e., Chicken *Gallus gallus domesticus* [8,20,23]). Thus, to fully recognize the variability and utility of body surface temperature as a proxy of the stress response in avian ecology, various taxonomical and ecological groups need to be examined. 

In this study, we used infrared thermography to examine surface body temperature changes in response to short-term stress, in a wild-living High-Arctic seabird, the Little Auk (or Dovekie) *Alle alle.* The Little Auk, being a little/medium-size seabird (body mass 140–180 g), represents a group of avian species for which stress-induced changes in body surface temperature have not yet been examined. Given the importance of the Little Auk in the Arctic food web [24,25], recognition of its stress response is crucial in studies on the species response to ongoing climate changes.

To simulate the stress situation we applied a standard capture-restrain protocol [26], where given parameters are measured twice in the same individuals: just after capturing (to establish a baseline) and after 30 min of restrain (to measure the response to the procedure). We measured two parameters: the eye-region temperature (hereafter TEYE) representing the body surface temperature and the corticosterone concentration in the blood (hereafter CORT) representing the stress level. As CORT is one of the glucocorticoid hormones released in response to a stressor [27,28], its simultaneous measurement with TEYE allowed us a reliable control of the stress level. 

We expected a significant difference between the baseline CORT/capture and handling stress-induced TEYE and the restrain stress-induced levels. Since various magnitudes and directions of TEYE stress responses in birds have been reported depending on various study designs and conditions (ambient temperature, stressor type, time scale, and bird size) [16,17,18,20], we expected a change in TEYE, but we did not formulate any directional hypothesis. For CORT, for which results are consistent among species, including the Little Auk [29], we expected a significant increase in the hormone level in reaction to the acute stress. We also investigated how TEYE and hormonal responses were related to each other. Since birds’ sex, body size, body reserves, and environmental factors (ambient temperature and humidity) may affect both the magnitude and the direction of CORT and/or the thermal stress response (as well as baseline/capture stress-induced and acute stress-induced parameters), we controlled all these variables in the analyses.

## 2. Materials and Methods

### 2.1. Study Species

The Little Auk is a zooplanktivorous alcid breeding colonially exclusively in the High Arctic. It nests in rock crevices located in the scree or under boulders of mountain slopes. The Little Auk female lays one egg annually incubated by both partners [24]. Incubation and chick-rearing duties are shared equally between the partners [30]. Both sexes are monomorphic in plumage, but males are often bigger than females (though there is a great overlap in measurements between the sexes) [31]. During incubation, the stress level expressed in the CORT level is similar in both sexes [32,33], but males have a tendency to exhibit a stronger CORT stress response than females [33].

### 2.2. Fieldwork

We performed our study in the large breeding colony of Little Auks at the Ariekammen slope in Hornsund, SW Spitsbergen (77°00′ N, 15°33′ E). It is considered as one of the largest breeding aggregation of the species in Svalbard [34].

We captured birds by hand while they were in the nest incubating the egg. During the whole procedure, while adults were handled and kept restrained (see below), the egg was kept in an insulated box to prevent it from cooling. Since the stage of the breeding, the sex of individual, and the time of the day can affect the body temperature and hormones’ concentrations, we sampled in both sexes during a short time interval in the specific phase of the breeding—mid incubation period (the second week; 3–4 and 7–11 July 2019). We also performed the fieldwork within a short time-window of the day (10:00–14:00) and similar weather conditions (no precipitation and no wind). Mid incubation is the period when birds are well after ovulation/spermatogenesis, so they are hormonally balanced. Besides, this period is also quite homogenous in terms of possible environmental stressors (for example, during the chick-rearing period, birds perform frequent foraging flights and so are more exposed to environmental effects). While aiming to sex-balance the sample size, which may otherwise be a challenge due to negligible sexual dimorphism [31], we captured at the nests both members of the pair. 

Following the standardized capture-restrain protocol [26], immediately after bird capture we collected an initial blood sample for CORT to establish its baseline level and then took a thermal photo to establish capture, handling, and blood sampling stress-induced TEYE. We did not consider this TEYE as a strict baseline because the thermal reaction may be as quick as 10 s after the stressor [15], but it served as a reference to the measurement taken at the second time-point of the protocol. We measured the duration of the blood sampling procedure precisely (mean duration = 2.1 min and min-max: 1.1–3.0 min), aiming to take the sample within the first 3 min following the moment of capture, as recommend by [26]. We took the first blood sample (~100 μL) from the right brachial vein in a heparinized capillary. After the initial blood sampling, we took thermal image. To do so, we focused on the bird’s head from the side (see details below). Then, we further handled the bird following a standard ornithological procedure (see details below). Completing all these activities, we kept the bird restrained in an opaque cloth bag for a period of 30 min, after which we took the second blood sample (~100 μL) from a brachial vein and the second thermal image (from the same head side)—to establish the acute stress-induced level of CORT and TEYE. After finishing all the procedures, we returned the bird and the egg to the nest.

The standard ornithological procedure implemented after the first session of bleeding and imaging included ringing (if the birds was not yet ringed), measuring the total head length (from the tip of bill to the end of skull, using a caliper accurate to 0.1 mm) and weighing using a PESOLA spring balance accurate to 1 g (PESOLA AG, Baar, Switzerland).

We took thermal photos of the bird’s head with a hand-held thermovision camera (FLIR E60, FLIR Systems Inc., Wilsonville, OR, USA, f = 18 mm, resolution 320 × 240, thermal sensitivity <0.05 °C) from a distance of 30–40 cm. As the body temperature and the loss of body heat could be affected by the ambient air temperature [17,18,35] and humidity [36,37], we measured these two parameters before taking thermal images using a hand-held Thermo-hygro-Barometer/logger D4130 (TEST-THERM, Kraków, Poland) device accurate to 0.4 °C and 2.5% of humidity. We included the ambient air temperature and the humidity in the models (see the details in the statistical analysis subsection).

While in the field (2–3 h) we kept the collected blood samples cool. After returning from the field, we then centrifugated them for 10 min at 6000 rpm. We kept frozen plasma and red cells separated (at −20 °C) and processed them in the laboratory (plasma—hormones assay; red cells—molecular sexing) within 4 months.

### 2.3. Processing of Thermal Images

**Figure 1:**
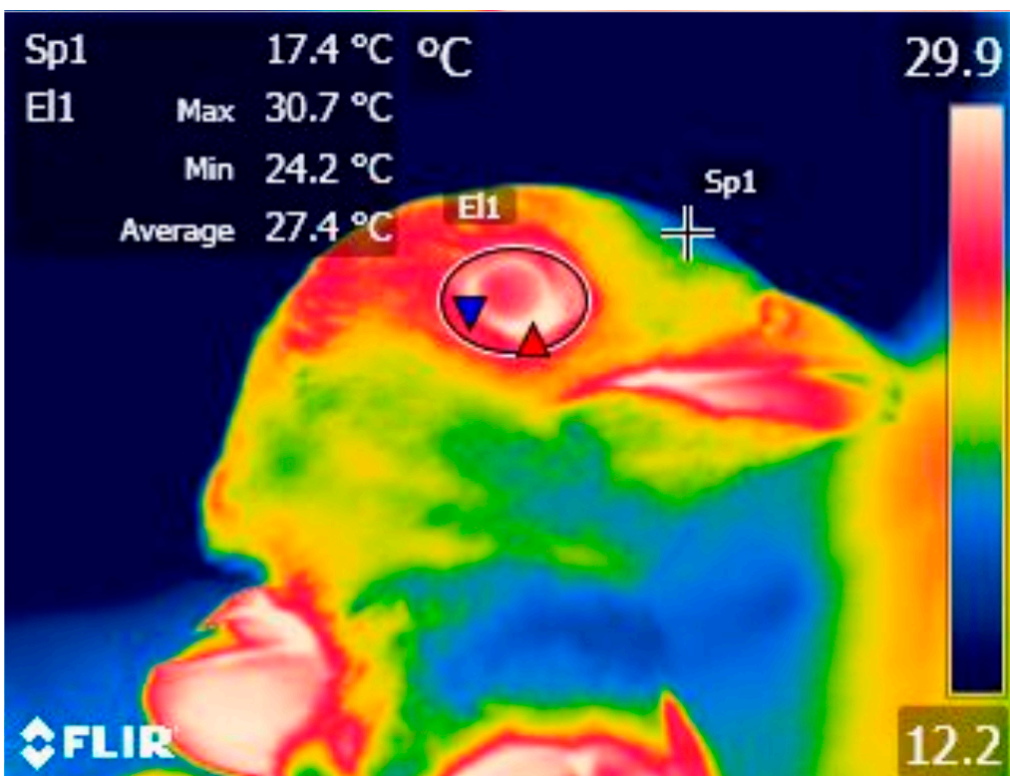
Example of thermal image processed in FLIR Tools+ software (FLIR Systems Inc., Wilsonville, OR, USA). Red and blue triangles indicate maximal and minimal temperature (°C), respectively, measured in the eye-region (delineated by a drawing tool ellipse). Sp1, El1 – the temperature measurement at the point and within ellipse, respectively, summarized in the top-left of the figure.

We measured the body surface temperature (°C) from the region of exposed skin around the eye (TEYE) in thermal images using FLIR Tools+ software (FLIR Systems Inc., Wilsonville, OR, USA). In thermo images we used a drawing tool in the software to delineate the eye-region with an ellipse (Figure 1). From this area we extracted the maximal TEYE, as the highest temperature measured from the eye region is assumed to be less susceptible to measurement error compared to mean values and is less likely to fluctuate according to the angle at which an individual is imaged [15,17,19]. The measured maximal TEYE represents the periorbital region [15,38].

For each individual we took several thermal images in a row (up to 8; mean ± SD: 3.1 ± 1.25 thermal images per individual per session) and took for analyses the highest maximal TEYE value recorded over all the images. The inter-image mean value of the standard deviation for maximal TEYE for all individuals was 1.13 for the first session and 1.11 for the second session.

### 2.4. Hormone Assay

We measured the baseline and stress-induced concentrations of CORT by radio-immunoassay at the Centre d’Etudes Biologiques de Chizé, France. We measured the total plasma CORT in the samples after ethyl ether extraction using a commercial antiserum, raised in rabbits against corticosterone 3-(O-carboxymethyl) oxime bovine serum albumin conjugate (Biogenesis, Poole, UK). We found that a cross-reaction was 9% with 1-desoxycorticosterone and <0.1% with other plasma steroids. We incubated duplicate aliquots of the extracts (100 μL) overnight at 4 °C with 8000 cpm of 3H-corticosterone (Amersham Pharmacia Biotech, Orsay, France) and antiserum. We separated the free and bound fractions of CORT by adding dextran-coated charcoal. After centrifugation, we counted the bound fraction in a liquid scintillation counter. We found that the minimal detectable CORT level was 0.3 ng. To measure intra-assay variation, we included four different samples ten times in the corticosterone assay. We found that the intra-assay variation for total corticosterone ranged from 5 to 12% (mean 6.7%).

### 2.5. Molecular Sexing

We performed molecular sexing at the University of Gdańsk, Poland. We extracted DNA from the frozen red cells using Blood Mini Kit (A&A Biotechnology, Gdynia, Poland). We amplified introns on the CHD-W and CHD-Z genes located on the avian sex chromosomes using the primers F2550 and R2718 [39] in PCR with an annealing temperature of 50 °C. We visualized the sex differences in the PCR products in UV-light on 1% agarose gel stained in Advanced Midori Green (Nippon Genetics Europe, Düren, Germany), with one band for male (i.e., ZZ) and two bands for female (i.e., ZW), both of the lengths being verified in respect to a standard ladder (100–1000 pb).

### 2.6. Data Analyses

We considered TEYE after the first session as a capture, handling, and blood sampling stress-induced temperature (hereafter handling stress-induced TEYE HSI) and the temperature after the second session as a restrain stress-induced temperature (TEYE RSI). The difference between TEYE HSI and TEYE RSI was then considered as a thermal stress response (TEYE SR).

For CORT, we considered its level after the first blood sampling session as the baseline level (CORT BL) as it was established on the basis of samples collected within a three minute time interval following the moment of capture [26]. The CORT level after the second blood session was considered as an acute stress-induced level (CORT ASI), and it was established on the basis of samples collected 30 min after the bird had been captured, during which time it was restrained [26,40]. The difference between CORT ASI and CORT BL was considered as a hormonal stress response (CORT SR).

Since heat production scales positively with body size [41], and can be reflected in body condition through changes in the metabolic rate made to protect energy reserves [19], we included birds’ body size and their body condition in our analyses. As a proxy of body size, we used the total head length. As a proxy of body condition, we used size-adjusted body, i.e., scaled mass index (SMI) using Formula (1) after [42]:

SMI = Mi × [Lo × Li^−1^]^bSMA^ (1)

where Mi = body mass of individual i; Li = linear body measurement of individual i (here total head length); bSMA = the scaling exponent estimated from the regression of M ~ L; Lo = arithmetic mean value of the linear measurement. We used a mean value of total head length as the linear body size measurement as it was significantly correlated with the body mass in adults (both sexes combined; Pearson correlation coefficient, r = 0.395, t_74_ = 3.695, *p* = 0.0004).

### 2.7. Statistical Analyses

Firstly, to investigate whether changes in the TEYE reflect response to the restrain procedure, we employed linear mixed models (LMM) with TEYE as a response variable. We started from a global model including the session (first/second sampling), the ambient air temperature (°C), the ambient humidity (%), and the sex (male/female) and all possible interactions between them as predictors, with the bird identity as a random factor. We performed parallelly two other global LMMs, with the same predictors but with sex exchanged with body size variable (total head length) or body condition proxy (scaled mass index). We could not pool all the predictors in the single model because of strong multicollinearity of body size and body condition with sex (variation inflation factor VIF > 155 in the LMM defined as TEYE ~ sex ∗ scaled mass index ∗ body size + (1|Bird ID)).

Then, to investigate whether TEYE and CORT change in a similar way in response to the procedure, we analyzed factors affecting the CORT level (response variable), starting with global LMM including the session (first/second sampling), the sex, the ambient air temperature, the TEYE, and all possible interactions between them as predictors, with the bird identity as a random factor. As for the TEYE models, we performed parallelly two other global LMMs with the same predictors except the sex was exchanged with the body size variable (total head length) or the body condition proxy (scaled mass index).

We used Akaike’s information criterion for small sample sizes (AICc) to select the best LMMs [43,44] with combinations of predictors included in the global model using the dredge function in the MuMIn package [45] in R software [46]. Due to a relatively small sample size, we reduced the number of terms in the candidate models following the “rule of thumb” of at least 10 events per candidate predictor [47], i.e., to N/10. We compared the relative performance of the models based on ΔAICc, i.e., the difference between the AIC value of the best model and the AIC value for each of the other models [43]. We presented only models with ΔAICc ≤ 2, suggested to be within the range of plausible models to best fit the observed data [43]. We selected as the best model the one with the lowest ΔAIC. To estimate the significance of the random effect in LMMs, we compared models with and without a random effect using the F test with the Kenward–Roger approximation [48].

Finally, to analyze factors affecting TEYE SR, we used linear models (LM). We started from a global model including sex, CORT BL, CORT SR, ambient air temperature during the first sampling session, and all possible interactions between them as predictors. For the same reasons as for the basic TEYE and CORT models, we performed parallelly two other global LMs with the same predictors but with sex exchanged with the body size variable (total head length) or the body condition proxy (scaled mass index); the multicollinearity of a model with all the predictors was high (variation inflation factor VIF > 155 in the LM defined as TEYE SR ~ sex ∗ scaled mass index ∗ body size).

In total, we captured, blood-sampled, and took thermal images of 41 individuals (i.e., 20 males and 21 females). However, as some blood samples were too small to analyze the CORT level and as we did not weigh a few individuals, the sample size was reduced to 35 individuals (17 females and 18 males) for CORT and to 33 individuals (17 females and 16 males) for the scaled mass index. See raw data in Supplementary Data File S1.

Before analyses we assessed whether the data sufficiently met the assumptions of the linear model using Q–Q plots (quantile expected in normal distribution versus quantile observed plot for residuals) and transformed the variable accordingly, if it did not meet the assumptions. We also checked the multicollinearity using the variation inflation factor and accepted only models with VIF < 5 [49]. Finally, we checked for heteroscedasticity of residuals on graphs (for LMMs) and using the Breush–Pagan test (for LMs) [50].

We performed LMMs in lme4, lmerTest [51], significance of the random effect in pbkrtest [48], model selection in MuMIn [45], variation inflation factor in car, and the Breush–Pagan test in lmtest [50] packages in R software [46].

## 3. Results

### 3.1. Factors Affecting Maximal Eye-Region Temperatures (TEYE)

**Figure 2:**
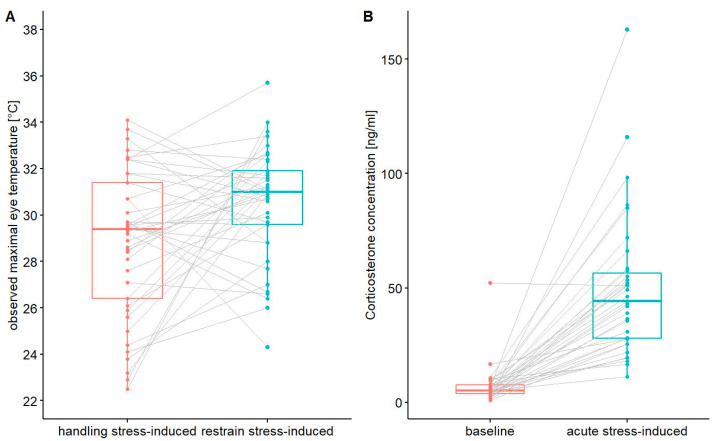
Comparison of maximal eye temperature of Little Auks between the first session (handling stress-induced; in red) and second session (restrain stress-induced; in blue) of the capture-restraint procedure (**A**) and corticosterone concentration in blood of Little Auks between the first session representing corticosterone baseline level and the second one representing the acute stress-induced level (**B**). Boxplots show the median (band inside the box), the first (25%) and third (75%) quartile (box), the lowest and the highest values within 1.5 interquartile range (whiskers), and outliers (dots); lines connect values recorded in the same individuals.

**Figure 3:**
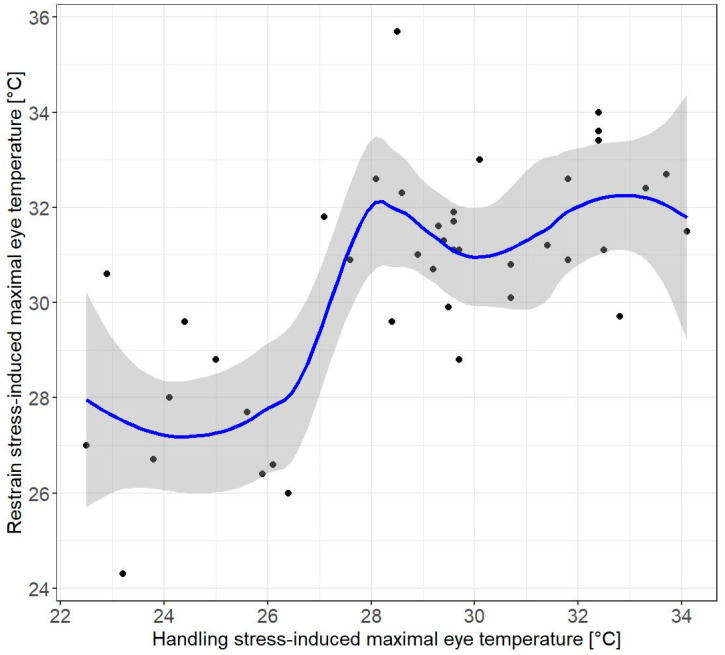
Relationship between handling, stress-induced eye-region temperature, and restrain stress-induced eye-region temperature. Smooth curve fitted by local polynomial regression (loess).

The highest-ranked LMMs with combinations of predictors included in the global models #1–3 describing TEYE included the session and the ambient air temperature (Table 1). Neither sex, body size, scaled mass index, nor humidity were present in the high-ranked models (Table 1). Consistently, TEYE recorded during the first session (HSI) was significantly lower (mean ± SD: 28.8 ± 3.17 °C, N = 41) compared to TEYE recorded during the second session (RSI) (mean ± SD: 30.5 ± 2.42 °C, N = 41 birds) (Table 2, Figure 2A). Besides, TEYE was positively related to the ambient temperature (Table 2). A random effect, bird identity, was significant (Table 2). We found that a relationship between TEYE HSI and TEYE RSI had an asymptotic pattern (Figure 3), suggesting a “ceiling effect,” i.e., the existence of a critical threshold of TEYE.

### 3.2. Factors Affecting Corticosterone Level (CORT)

The highest-ranked LMMs with combinations of predictors included in the global models #4–6 describing CORT level included only one predictor, session (Table 1). Neither the sex, the body size, nor the scaled mass index were present in the high-ranked models (Table 1). The CORT level in the first session (baseline, mean ± SD: 6.96 ± 8.51 ng/mL, N = 35) was significantly lower compared to the second session (acute stress-induced, mean ± SD: 49.3 ± 31.3 ng/mL, N = 35 birds); (Table 2, Figure 2B). A random effect, bird identity, was significant (Table 2).

### 3.3. Factors Affecting Thermal Stress Response (TEYE SR)

The highest-ranked LMs with combinations of predictors included in the global models #1–3 describing TEYE SR included only intercept (Table 3). The second highest-ranked models included CORT SR (for global models #1 and #3) or body size (model #2). In all these models, mentioned predictors were not significant (Table 4).

## 4. Discussion

Applying a standard capture-restrain protocol (as recommended by [26]) in the Little Auks, we measured birds’ thermal stress response. As expected, birds stressed with the procedure (i.e., the level of the corticosterone increased considerably), and so their body temperature increased in response to the stressful situation. Our findings indicate potential of thermography in avian ecophysiology and that could be applicable also in seabirds (so far used mainly in passerines, Galliformes, and pigeons).

### 4.1. Factors Affecting Maximal Eye-Region Temperatures

We found that both maximal eye temperature (TEYE) and corticosterone (CORT) were affected by session, increasing between the first and the second session of the applied protocol. Since the stress reaction is associated with an increase in CORT [27] and other factors affecting the CORT level [52,53,54,55] were similar (phase of breeding, circadian rhythm) or included in analyses (sex, weather), our results strongly suggest that a significant increase in TEYE and CORT indicate a reaction to stress. As expected, we found a positive relationship between TEYE and ambient temperature regardless of the blood sampling session. This result is consistent with other studies showing surface temperature to be sensitive to environmental conditions [18,19,56].

Interestingly, we found a significant effect of bird identity (employed as a random effect in LMMs) on TEYE resulting in various directions and/or strengths of TEYE SR. Individual variation in the magnitude of avian stress-induced thermal response has been already reported [21,57]. Although the cause of such variation is unknown, it cannot be excluded that the degree to which handling elicited a physiological stress response differed among studied individuals [21]. Overall, this inter-individual variation both in the CORT and the TEYE stress response does not affect the general conclusions but may be interesting on its own and would be worthy of a separate study.

### 4.2. Magnitude and Direction of Thermal Response to Acute Stress

The relationship between the TEYE levels in the two sessions (i.e., handling vs. restrain) observed in our study followed an asymptotic pattern (Figure 3) indicating an increase in TEYE until some threshold. It suggests a “ceiling effect” as body temperature regulation in birds and mammals is rather tight [7]. Particularly, strong and/or long-lasting temperature increases may cause serious damage that is life threatening for animals and humans [58,59]. A similar pattern has been reported in the combs surface temperature of chickens. Weighed hens (i.e., less stressed) showed greater increases in the comb temperature and the foraging rate between undisturbed and post-handling days than blood-sampled hens (i.e., more stressed). It may be interpreted by higher “baseline” (undisturbed) stress levels in blood-sampled hens, reducing the scope for an increase [20].

The direction of TEYE SR observed in Little Auks, i.e., an increase in TEYE between the two sessions, was consistent with changes observed in other medium-sized birds. In the Svalbard Ptarmigan *Lagopus muta hyperborea,* for example, the maximum head surface temperature increased by an average of 0.67 °C in response to a stressor [35]. However, in smaller species like passerines, the opposite thermal stress reaction was observed, e.g., in Blue Tits, TEYE decreased by 2 °C [15].

Similar discrepancies in the direction of thermal change have been observed in the case of core body temperature. In Mallards *Anas platyrhynchos*, a restraint protocol induced an increase of 0.5 °C in the core body temperature [10]. Similarly, Rock Pigeons *Columbia livia* responded to the transfer to a new cage by a 0.44 °C increase in the core body temperature [11]. In Common Eiders *Somateria mollissima* handling resulted in a 2 °C increase in cloacal temperature [9]. In smaller species however, thermal change had the opposite direction. In Barn Swallows *Hirundo rustica*, a short-time handling resulted in a 0.27 °C decrease in cloacal temperature [60]. In Great Tits, a restraint protocol induced a 0.5–0.6 °C decrease in cloacal temperature [57].

These divergent thermal responses in smaller and larger avian species may be explained, if not apart from differences in experimental design, by the body size as well as the ambient temperature. Smaller species having higher surface-area-to-volume ratios are prone to greater heat transfer to the environment per unit volume [16]. Moreover, stress-related hypothermia in small birds (with inherently higher thermal conductance) handled below their thermoneutral zone may be explained either by an increased rate of heat-loss due to conductive cooling from cold hands, decreased insulation from compression of plumage and prevention of ptiloerection, or by reduced heat production, if handling causes tonic immobility-preventing activity/thermoregulatory heat substitution [35,61,62].

Different directions of thermal responses in various species and group of individuals within one species may also be explained by different time scales and dynamics of recorded reaction. In Blue Tits, TEYE dropped within 10 s after closing the trap gate (stressor used in the study) [15]. In another study covering a longer time-span, the TEYE rapidly dropped below the baseline in trapped, handled, and blood-sampled Blue Tits and then recovered above baseline before declining again but more slowly until the end of the test, 160 s after trap closure [16]. In the chicken, TEYE declined at the short-term scale (2–2.5 h) but increased at the longer-term scale (few days) after a stress was induced (enrichment withdrawal) [20]. Thus, it is important to consider time scale when comparing the results of different studies.

Ambient temperatures may also affect the strength and/or direction of thermal reaction. In Svalbard Ptarmigans core temperature increased less, and back skin temperature decreased more at −20 °C ambient temperature than it did at 0 °C [35]. Black-capped chickadees exposed to rotating stressors reduced TEYE and dry heat loss when held below thermoneutral temperatures but increased TEYE and dry heat loss when held above thermoneutral temperatures compared with controls; in the thermoneutral zone, TEYE increased with ambient temperature both in stressed and control individuals [17]. These differences in direction of the thermal reaction in various ambient temperatures may play a functional role. They may be explained by the thermoprotective hypothesis, saying that changes in body surface temperature after exposure to stressors reduce energetic costs incurred during activation of a stress response, by promoting heat conservation at low temperatures (conservatively, below thermoneutrality) and heat dissipation at high temperature (conservatively, above thermoneutrality) [17]. Keeping high temperature in the eye region in response to acute stress may have additional functional value for visual acuity, crucial to follow predator movements and/or to maintain foraging efficiency in a visually guided bird [18] like the Little Auk.

Ambient temperatures recorded during our study (mean 10–11 °C) were relatively high compared to the multi-year (1979–2017) mean for July (4.5 °C; data from the meteorological station in Polish Polar Station Hornsund (Meteorological Bulletin of Polish Polar Station in Hornsund), 900 m from the colony), which may have affected both the strength and the direction of the observed thermal response. Further studies performed in various ambient temperatures are needed to fully comprehend the pattern of Little Auks’ thermal reaction to stress.

Arctic birds are highly adapted to cold environments, and the physiological mechanisms enhancing cold tolerance may increase thermal sensitivity and reduce thermoregulatory capacity at warmer temperatures [63]. A rapid climate increase in the Arctic (air temperature increase being two to three times higher than the global increase [64,65]) may challenge physiological capacity to tolerate warmer temperatures in well-insulated High-Arctic endotherms. Given the range of the Little Auk thermoneutral zone (from 4.5 to ~20 °C [66]), and a positive relationship between TEYE and ambient air temperature, one may expect that a hypothermal reaction to acute stress may lead to short-term overheating of well-insulated species like the Little Auk. A high increase in temperature may cause serious damage that is life threatening for animals [58,59]. Studies on another alcid species, the Brünnich’s Guillemot *Uria lomvia*, revealed that the highest rates of water loss to combat overheating occurred when the ambient temperature was above 17 °C [67]. Thus, heat stress may be one of the consequences of global changes for Little Auks, next to deterioration of foraging conditions [68] and increased exposure to mercury contamination [69,70].

### 4.3. Thermal vs. Hormonal Response to Acute Stress

Interestingly, despite directional similarity between thermal and hormonal responses, we did not find a significant relationship between TEYE SR and CORT BL. This finding is concordant with results of the recent experimental study on the House Sparrow *Passer domesticus* showing that changes in skin temperature recorded by infrared thermography reflects the reactivity of the hypothalamic–pituitary–adrenal (HPA) axis controlling the stress response but is not a good proxy for natural variation in circulating glucocorticoid levels [71]. As mentioned before, we controlled some other factors affecting the CORT level by performing the study in the short period of time and accounting for sex and weather conditions in the analyses. However, we detected some inter-individual variety in CORT BL. We detected two individuals with very high CORT BL (Figure 2B). It cannot be excluded that these individuals were under a chronic stress. It has been found that Little Auks with high baseline CORT were characterized by an attenuated CORT stress response [33]. Thus, mentioned inter-individual variety in CORT BL together with the “ceiling effect” (increasing in TEYE until some threshold) may have resulted in a lack of a significant relationship between CORT BL and TEYE SR.

We did not find a significant effect of sex on either TEYE or TEYE SR, which may be explained by similar body size in both sexes [31] and the presence of brood patches in both sexes. Heat transfer from the parental body to the egg may affect the heat dissipation capacity and the surface temperature of an individual, which may affect males and females differently in species with uniparental incubation [17]. The lack of a significant effect of body size and body condition variables on TEYE SR may be explained in terms of good insulation of all individuals despite differences in body size or body condition and/or a small range of variation in body size and condition within the studied individuals.

### 4.4. Implementation of Infrared Thermography in the Field Studies

Our results demonstrate that infrared thermography may serve as a non-invasive and fast measure of the activation of the HPA axis controlling the stress response in a small diving seabird in the Arctic field conditions. This opens a new perspective for implementation of this proxy of stress level in ecological studies. The infrared thermography is still not a cheap technique considering the costs of a thermal camera with good resolution. However, if the purchase of this equipment is affordable, stress level estimation with infrared thermography has the advantage of being non-invasive compared to other techniques to measure stress requiring blood sampling, such as the corticosterone level and/or the heterophils-to-leucocytes ratio [14]. Moreover, thermal imagery analyses may be done in intuitive software provided with the camera. There are some limitations of the thermal imagery and the protocol tested here, however. First, the pace of thermal reaction differs from hormonal reaction. It is known that the CORT level in birds rises after ~3 min after capture and hits a peak after 30 min [26,40]. However, the TEYE reaction in birds is less recognized. We showed changes in TEYE for a ~30 min period, already demonstrating the method utility, but the dynamics of the thermal stress response (especially from the no-stress point before capture) deserves further research. Given a possible faster thermal response to stressors and many stressors involved during capturing, handling, and blood sampling, it would be better to take thermal images just after bird capture. In our case, it was not possible given the necessity of very fast blood sampling, securing an estimation of the baseline CORT level. Another limitation of the method we tested here is that it is still uncertain how universal are the results of our study. Given that changes in body surface temperature should promote heat conservation at low temperatures, and heat dissipation at high temperatures (thermoprotective hypothesis, [17]), the direction of change in the eye-region temperature may depend on the ambient temperature. However, it is likely that, given similar patterns of activity-thermoregulatory heat substitution in endotherms living in cold environments [62], at least polar seabirds should respond in a similar way. Finally, recent studies have demonstrated that the surface temperature of an object perceived by the thermal camera can vary according to the distance and the angle of incidence in an infrared thermography image [38]. Thus, changes in the relative orientation of an object during infrared thermography imaging may conceal or distort true changes in the surface temperature [21].

## 5. Conclusions

Our study revealed a significant increase in TEYE and CORT in a High-Arctic seabird, the Little Auk, in response to acute stress (capture-restrain procedure). The increase in TEYE and CORT were not correlated, however. All this suggests that thermography may be a useful tool in measuring the hypothalamic–pituitary–adrenal (HPA) axis reactivity under acute activation in small/medium-size polar seabirds, such as the Little Auk. The hyperthermic stress reaction in well insulated Arctic birds may be challenged by the ongoing and expected climate change-driven increase in air temperature. To fully understand the mechanisms of thermal stress reaction in birds, studies on other avian groups, living in various habitats and climatic conditions are needed.

## Figures and Tables

**Table 1:** Rank of the highest-ranked linear mixed models within global models estimating the effects of various predictors on maximal eye temperature (TEYE) and corticosterone level (CORT). AMBT—ambient air temperature, BodySize—total head length, BodyCond—scaled body mass, and Int—intercept. Only the best models with ΔAICc ≤ 2 are presented; Akaike’s weights were calculated from the full set of models. N—number of the studied individuals, df – degrees of freedom, logLik—log-likelihood, AICc—Second-order Akaike Information Criterion, **ΔAICc**—difference between the best model (with smallest AICc) and each model.

Model Parameters	df	logLik	AICc	ΔAICc	Akaike’s Weights
global model #1: TEYE ~ Session ∗ Sex ∗ AMBT ∗ Humidity + (1|Bird ID), N = 41					
Int + AMBT + Session + (1|Bird ID)	5	−182.189	375.2	0.00	0.530
Int + AMBT + Session + Sex + (1|Bird ID)	6	−181.684	376.5	1.32	0.274
Int + AMBT + (1|Bird ID)	4	−184.323	377.2	2.00	0.195
global model #2: TEYE ~ Session ∗ BodySize ∗ AMBT ∗ Humidity + (1|Bird ID), N = 41					
Int + AMBT + Session + (1|Bird ID)	5	−182.189	375.2	0.00	0.564
Int + AMBT + Session + BodySize + (1|Bird ID)	6	−181.932	377.0	1.81	0.228
Int + AMBT + (1|Bird ID)	4	−184.323	377.2	2.00	0.208
global model #3: TEYE ~ Session ∗ BodyCond ∗ AMBT ∗ Humidity + (1|Bird ID), N = 38					
Int + AMBT + Session + (1|Bird ID)	5	−169.920	350.7	0.0	0.701
Int + AMBT + (1|Bird ID)	4	−171.919	352.4	1.7	0.299
global model #4: log(CORT) ~ Session ∗ Sex ∗ TEYE ∗ AMBT + (1|Bird ID), N = 35					
Int + Session + (1|Bird ID)	4	−73.448	155.5	0.00	1.000
global model #5: log(CORT) ~ Session ∗ BodySize ∗ TEYE ∗ AMBT + (1|Bird ID), N = 35					
Int + Session + (1|Bird ID)	4	−73.448	155.5	0.00	1.000
global model #6: log(CORT) ~ Session ∗ BodyCond ∗ TEYE ∗ AMBT + (1|Bird ID), N = 33					
Int + Session + (1|Bird ID)	4	−69.197	147.0	0.00	1.000

**Table 2:** The highest-ranked LMMs with combinations of predictors included in the global models listed in Table 1 estimating the effects of various predictors on eye region temperature (TEYE) and corticosterone level (CORT) in Little Auks in capture-restraint experiment. Predictors: Session (first representing handling stress-induced TEYE and baseline CORT, and the second representing restrain-induced TEYE and acute stress-induced CORT), AMBT—ambient air temperature, Bird ID r.e.—bird identity (random effect). Significance of a random effect, the bird identity (Bird ID) estimated by F-test with Kenward–Roger approximation. R^2^c—conditional R squared—a variance explained by the entire model, including both fixed and random effects [45], df – degrees of freedom.

Response Variable	Predictor	Estimate	SE	df	t	*p*
The highest-ranked model within the global LMM #1, R^2^c = 0.616						
TEYE	Intercept	22.895	1.298	60.268	17.636	<0.001
	Session	0.887	0.423	47.841	2.100	0.0411
	AMBT	0.600	0.125	59.838	4.782	<0.001
	Bird ID r.e.	19.851	-	2	-	<0.001
The highest-ranked model within the global LMM #4, R^2^c = 0.712						
log(CORT)	Intercept	1.620	0.114	68	14.17	<0.001
	Session	2.111	0.162	68	13.06	<0.001
	Bird ID r.e.	170.6	-	1		<0.001

**Table 3:** Rank of the highest-ranked linear models within global models estimating the effects of various predictors on thermal (TEYE SR) or hormonal (CORT SR) stress response. Codes: AMBT BL—baseline ambient air temperature, BodySize—total head length, BodyCond—scaled mass index, Int—intercept, and CORT BL—corticosterone baseline level. Only the best models with ΔAICc ≤ 2 are presented; Akaike’s weights were calculated from the full set of models. df – degrees of freedom, logLik—log-likelihood, AICc—Second-order Akaike Information Criterion, ΔAICc—difference between the best model (with smallest AICc) and each model.

Model Parameters	df	logLik	AICc	ΔAICc	Akaike’s Weights
global model #1: TEYE SR ~ Sex ∗ CORT SR ∗ CORT BL ∗ AMBT BL, N = 35					
Int	2	−78.810	162.0	0.00	0.527
Int + CORT SR	3	−78.308	163.4	1.39	0.262
Int + AMBT BL	3	−78.526	163.8	1.83	0.211
global model #2: TEYE SR ~ BodySize ∗ CORT SR ∗ CORT BL ∗ AMBT BL, N = 35					
Int	2	−78.810	162.0	0.00	0.410
Int + BodySize	3	−78.228	163.2	1.23	0.221
Int + CORT SR	3	−78.308	163.4	1.39	0.204
Int + CORT BL	3	−78.526	163.8	1.83	0.164
global model #3: TEYE SR ~ log(BodyCond) ∗ CORT SR ∗ CORT BL ∗ AMBT BL, N = 32					
Int	2	−72.115	148.6	0.00	0.552
Int + CORT SR	3	−71.727	150.3	1.67	0.240
Int + CORT BL	3	−71.865	150.6	1.94	0.209

**Table 4:** The highest-ranked linear models with combinations of predictors included in the global models listed in Table 3 estimating the effects of various predictors on thermal stress response (eye-region temperature—TEYE SR) in Little Auks in capture-restraint experiment. CORT SR—corticosterone stress response and BodySize—total head length. SE—standard error, df—degrees of freedom.

Response Variable	Predictor	Estimate	SE	df	F	*p*
The second-highest ranked model within the global LM#1, R^2^ = 0.028						
TEYE SR	CORT SR	−0.012	0.012	1	0.961	0.334
The second-highest ranked model within the global LM#2, R^2^ = 0.033						
TEYE SR	BodySize	−15.980	16.770	1	1.117	0.298
The second-highest ranked model within the global LM#3, R^2^ = 0.024						
TEYE SR	CORT SR	−0.010	0.012	1	0.738	0.397

## Data Availability

The dataset supporting this article has been uploaded as part of the electronic supplementary material.

## Supplementary Materials

The following supporting information can be downloaded at: https://www.mdpi.com/article/10.3390/ani12040499/s1, File S1: Supplementary Data File with the results of all experiments.

## References

[1] Romero M., Wingfield J. (2015). Tempests, Poxes, Predators, and People: Stress in Wild Animals and How They Cope.

[2] McEwen B.S., Wingfield J.C. (2003). The concept of allostasis in biology and biomedicine. Horm. Behav..

[3] Wingfield J.C., Romero L.M. (2001). Adrenocortical Responses to Stress and Their Modulation in Free-Living Vertebrates. Comprehensive Physiology.

[4] Sapolsky R.M., Romero L.M., Munck A.U. (2000). How Do Glucocorticoids Influence Stress Responses? Integrating Permissive, Suppressive, Stimulatory, and Preparative Actions. Endocr. Rev..

[5] Wingfield J.C. (2003). Control of behavioural strategies for capricious environments. Anim. Behav..

[6] Oka T., Oka K., Hori T. (2001). Mechanisms and Mediators of Psychological Stress-Induced Rise in Core Temperature. Psychosom. Med..

[7] Bouwknecht A.J., Olivier B., Paylor R.E. (2007). The stress-induced hyperthermia paradigm as a physiological animal model for anxiety: A review of pharmacological and genetic studies in the mouse. Neurosci. Biobehav. Rev..

[8] Herborn K.A., Graves J.L., Jerem P., Evans N.P., Nager R., McCafferty D.J., McKeegan D.E.F. (2015). Skin temperature reveals the intensity of acute stress. Physiol. Behav..

[9] Cabanac A.J., Guillemette M. (2001). Temperature and heart rate as stress indicators of handled common eider. Physiol. Behav..

[10] Gray D.A., Maloney S.K., Kamerman P.R. (2008). Restraint increases afebrile body temperature but attenuates fever in Pekin ducks (*Anas platyrhynchos*). Am. J. Physiol.-Regul. Integr. Comp. Physiol..

[11] de Aguiar Bittencourt M., Melleu F.F., Marino-Neto J. (2015). Stress-induced core temperature changes in pigeons (*Columba livia*). Physiol. Behav..

[12] Foster S., Ijichi C. (2017). The association between infrared thermal imagery of core eye temperature, personality, age and housing in cats. Appl. Anim. Behav. Sci..

[13] Vinkers C.H., Penning R., Hellhammer J., Verster J.C., Klaessens J.H.G.M., Olivier B., Kalkman C.J. (2013). The effect of stress on core and peripheral body temperature in humans. Stress.

[14] Gormally B.M.G., Romero L.M. (2020). What are you actually measuring? A review of techniques that integrate the stress response on distinct time-scales. Funct. Ecol..

[15] Jerem P., Herborn K., McCafferty D., McKeegan D., Nager R. (2015). Thermal imaging to study stress non-invasively in unrestrained birds. J. Vis. Exp..

[16] Jerem P., Jenni-Eiermann S., McKeegan D., McCafferty D.J., Nager R.G. (2019). Eye region surface temperature dynamics during acute stress relate to baseline glucocorticoids independently of environmental conditions. Physiol. Behav..

[17] Robertson J.K., Mastromonaco G., Burness G. (2020). Evidence that stress-induced changes in surface temperature serve a thermoregulatory function. J. Exp. Biol..

[18] Winder L.A., White S.A., Nord A., Helm B., McCafferty D.J. (2020). Body surface temperature responses to food restriction in wild and captive great tits. J. Exp. Biol..

[19] Jerem P., Jenni-Eiermann S., Herborn K., McKeegan D., McCafferty D.J., Nager R.G. (2018). Eye region surface temperature reflects both energy reserves and circulating glucocorticoids in a wild bird. Sci. Rep..

[20] Herborn K.A., Jerem P., Nager R.G., McKeegan D.E.F., McCafferty D.J. (2018). Surface temperature elevated by chronic and intermittent stress. Physiol. Behav..

[21] Tabh J.K.R., Burness G., Wearing O.H., Tattersall G.J., Mastromonaco G.F. (2021). Infrared thermography as a technique to measure physiological stress in birds: Body region and image angle matter. Physiol. Rep..

[22] Davis A.K., Maney D.L., Maerz J.C. (2008). The use of leukocyte profiles to measure stress in vertebrates: A review for ecologists. Funct. Ecol..

[23] Edgar J.L., Nicol C.J., Pugh C.A., Paul E.S. (2013). Surface temperature changes in response to handling in domestic chickens. Physiol. Behav..

[24] Stempniewicz L. (2001). BWP update. Little Auk (*Alle alle*). J. Birds West. Palearct..

[25] Wojczulanis-Jakubas K., Jakubas D., Stempniewicz L. (2022). The Little Auk Alle alle: An ecological indicator of a changing Arctic and a model organism. Polar Biol..

[26] Wingfield J.C., Deviche P., Sharbaugh S., Astheimer L.B., Holberton R., Suydam R., Hunt K. (1994). Seasonal changes of the adrenocortical responses to stress in redpolls, *Acanthis flammea*, in Alaska. J. Exp. Zool..

[27] Johnstone C.P., Reina R.D., Lill A. (2012). Interpreting indices of physiological stress in free-living vertebrates. J. Comp. Physiol. B Biochem. Syst. Environ. Physiol..

[28] Romero L.M. (2004). Physiological stress in ecology: Lessons from biomedical research. Trends Ecol. Evol..

[29] Wojczulanis-Jakubas K., Jakubas D., Chastel O. (2013). Behavioural and hormonal stress responses during chick rearing do not predict brood desertion by female in a small Arctic seabird. Horm. Behav..

[30] Wojczulanis-Jakubas K., Jakubas D., Stempniewicz L. (2009). Sex-specific parental care by incubating little auks (*Alle alle*). Ornis Fenn..

[31] Jakubas D., Wojczulanis K. (2007). Predicting the Sex of Dovekies by Discriminant Analysis. Waterbirds.

[32] Wojczulanis-Jakubas K., Jakubas D., Chastel O., Kulaszewicz I. (2015). A big storm in a small body: Seasonal changes in body mass, hormone concentrations and leukocyte profile in the little auk (*Alle alle*). Polar Biol..

[33] Wojczulanis-Jakubas K., Jakubas D., Kulpińska-Chamera M., Chastel O. (2018). Sex- and breeding stage-specific hormonal stress response of seabird parents. Horm. Behav..

[34] Keslinka L.K., Wojczulanis-Jakubas K., Jakubas D., Neubauer G. (2019). Determinants of the little auk (*Alle alle*) breeding colony location and size in W and NW coast of Spitsbergen. PLoS ONE.

[35] Nord A., Folkow L.P. (2019). Ambient temperature effects on stress-induced hyperthermia in Svalbard ptarmigan. Biol. Open.

[36] Lin H., Zhang H.F., Du R., Gu X.H., Zhang Z.Y., Buyse J., Decuypere E. (2005). Thermoregulation responses of broiler chickens to humidity at different ambient temperatures. II. Four weeks of age. Poult. Sci..

[37] Tsilingiris P.T. (2008). Thermophysical and transport properties of humid air at temperature range between 0 and 100 °C. Energy Convers. Manag..

[38] Playà-Montmany N., Tattersall G.J. (2021). Spot size, distance and emissivity errors in field applications of infrared thermography. Methods Ecol. Evol..

[39] Griffiths R., Double M.C., Orr K., Dawson R.J.G. (1998). A DNA test to sex most birds. Mol. Ecol..

[40] Pakkala J.J., Ryan Norris D., Newman A.E.M. (2013). An experimental test of the capture-restraint protocol for estimating the acute stress response. Physiol. Biochem. Zool..

[41] Blackburn T.M., Gaston K.J., Loder N. (1999). Geographic gradients in body size: A clarification of Bergmann’s rule. Divers. Distrib..

[42] Peig J., Green A.J. (2009). New perspectives for estimating body condition from mass/length data: The scaled mass index as an alternative method. Oikos.

[43] Burnham K.P., Anderson D.R. (2002). Model Selection and Multimodel Inference. A Practical Information-Theoretic Approach.

[44] Hegyi G., Garamszegi L.Z. (2011). Using information theory as a substitute for stepwise regression in ecology and behavior. Behav. Ecol. Sociobiol..

[45] Bartoń K. MuMIn: Multi-Model Inference. R Package Version 1.10.0. 2017. https://r-forge.r-project.org/projects/mumin/.

[46] R Core Team (2020). R: A Language and Environment for Statistical Computing.

[47] Peduzzi P., Concato J., Feinstein A.R., Holford T.R. (1995). Importance of events per independent variable in proportional hazards regression analysis II. Accuracy and precision of regression estimates. J. Clin. Epidemiol..

[48] Halekoh U., Højsgaard S. (2014). A kenward-Roger approximation and parametric bootstrap methods for tests in linear mixed models-the R package pbkrtest. J. Stat. Softw..

[49] Zuur A.F., Ieno E.N., Elphick C.S. (2010). A protocol for data exploration to avoid common statistical problems. Methods Ecol. Evol..

[50] Zeileis A., Hothorn T. (2002). Diagnostic Checking in Regression Relationships. R News.

[51] Kuznetsova A., Brockhoff P.B., Christensen R.H.B. (2017). lmerTest Package: Tests in Linear Mixed Effects Models. J. Stat. Softw..

[52] Romero L.M., Soma K.K., Wingfield J.C. (1998). Hypothalamic-pituitary-adrenal axis changes allow seasonal modulation of corticosterone in a bird. Am. J. Physiol.-Regul. Integr. Comp. Physiol..

[53] Romero L.M. (2002). Seasonal changes in plasma glucocorticoid concentrations in free-living vertebrates. Gen. Comp. Endocrinol..

[54] Goymann W., Trappschuh M., Urasa F. (2017). Corticosterone concentrations reflect parental expenditure in contrasting mating systems of two coucal species. Front. Ecol. Evol..

[55] Jenni-Eiermann S., Glaus E., Grüebler M., Schwabl H., Jenni L. (2008). Glucocorticoid response to food availability in breeding barn swallows (Hirundo rustica). Gen. Comp. Endocrinol..

[56] Hill R.W., Beaver D.L., Veghte J.H. (1980). Body Surface Temperatures and Thermoregulation in the Black-Capped Chickadee (*Parus atricapillus*). Physiol. Zool..

[57] Carere C., Van Oers K. (2004). Shy and bold great tits (*Parus major*): Body temperature and breath rate in response to handling stress. Physiol. Behav..

[58] Sharma H.S., Hoopes P.J. (2009). Hyperthermia induced pathophysiology of the central nervous system. Int. J. Hyperth..

[59] Sundgren-Andersson A.K., Östlund P., Bartfai T. (1998). Simultaneous Measurement of Brain and Core Temperature in the Rat during Fever, Hyperthermia, Hypothermia and Sleep. Neuroimmunomodulation.

[60] Møller A.P. (2010). Body temperature and fever in a free-living bird. Comp. Biochem. Physiol.-B Biochem. Mol. Biol..

[61] Lewden A., Nord A., Petit M., Vézina F. (2017). Body temperature responses to handling stress in wintering Black-capped Chickadees (*Poecile atricapillus* L.). Physiol. Behav..

[62] Humphries M.M., Careau V. (2011). Heat for nothing or activity for free? Evidence and implications of activity-thermoregulatory heat substitution. Proceedings of the Integrative and Comparative Biology.

[63] O’Connor R.S., Le Pogam A., Young K.G., Robitaille F., Choy E.S., Love O.P., Elliott K.H., Hargreaves A.L., Berteaux D., Tam A. (2021). Limited heat tolerance in an Arctic passerine: Thermoregulatory implications for cold-specialized birds in a rapidly warming world. Ecol. Evol..

[64] Box J.E., Colgan W.T., Christensen T.R., Schmidt N.M., Lund M., Parmentier F.J.W., Brown R., Bhatt U.S., Euskirchen E.S., Romanovsky V.E. (2019). Key indicators of Arctic climate change: 1971–2017. Environ. Res. Lett..

[65] Cohen J., Zhang X., Francis J., Jung T., Kwok R., Overland J., Ballinger T.J., Bhatt U.S., Chen H.W., Coumou D. (2020). Divergent Consensuses on Arctic Amplification Influence on Midlatitude Severe Winter Weather.

[66] Gabrielsen G.W., Taylor J.R.E., Konarzewski M., Mehlum F. (1991). Field and Laboratory Metabolism and Thermoregulation in Dovekies (*Alle alle*). Auk.

[67] Gaston A.J., Elliott K.H. (2013). Effects of climate-induced changes in parasitism, predation and Predator-Predator interactions on reproduction and survival of an Arctic Marine Bird. Arctic.

[68] Jakubas D., Wojczulanis-Jakubas K., Iliszko L.M., Strøm H., Stempniewicz L. (2017). Habitat foraging niche of a High Arctic zooplanktivorous seabird in a changing environment. Sci. Rep..

[69] Stern G.A., Macdonald R.W., Outridge P.M., Wilson S., Chételat J., Cole A., Hintelmann H., Loseto L.L., Steffen A., Wang F. (2012). How does climate change influence arctic mercury?. Sci. Total Environ..

[70] Fort J., Robertson G.J., Grémillet D., Traisnel G., Bustamante P. (2014). Spatial Ecotoxicology: Migratory Arctic Seabirds Are Exposed to Mercury Contamination While Overwintering in the Northwest Atlantic. Environ. Sci. Technol..

[71] Ouyang J.Q., Macaballug P., Chen H., Hodach K., Tang S., Francis J.S. (2020). Infrared thermography is an effective, noninvasive measure of HPA activation. Stress.

[72] Buchanan K.L., Burt de Perera T., Carere C., Carter T., Hailey A., Hubreacht R., Jenning D.J., Metcalfe N.B., Pitcher T.E., Péron F. (2012). Guidelines for the Use of Animals Guidelines for the treatment of animals in behavioural research and teaching. Anim. Behav..

[73] Kilkenny C., Browne W.J., Cuthill I.C., Emerson M., Altman D.G. (2010). Improving Bioscience Research Reporting: The ARRIVE Guidelines for Reporting Animal Research. PLOS Biol..

